# ‘Building bridges’: reflections and recommendations for co-producing health research

**DOI:** 10.1186/s40900-023-00528-0

**Published:** 2023-12-06

**Authors:** Vasiliki Papageorgiou, Lindsay H. Dewa, Jane Bruton, Keitumetse-Kabelo Murray, Nick Hewlett, Wezi Thamm, Husseina Hamza, Pino Frumiento, Robyn Steward, Melissa Bradshaw, Ellie Brooks-Hall, Silvia Petretti, Sarah Ewans, Mark Williams, Dorota Chapko

**Affiliations:** 1 NIHR Imperial Biomedical Research Centre Patient Experience Research Centre, Imperial College London, London, UK; 2 NIHR Imperial Biomedical Research Centre, Institute of Global Health Innovation, Imperial College London, London, UK; 3 NIHR Applied Research Collaboration Northwest London, Imperial College London, London, UK; 4 Positively UK, London, UK; 5 Heart N Soul, London, UK

**Keywords:** Co-production, Community participation, COVID-19, Disability, HIV, Mental health

## Abstract

**Background:**

Co-produced research is when all stakeholders, including experts by experience and researchers, work together to conceptualise, design, deliver and disseminate research to enhance understanding and knowledge. This type of participatory inquiry is being increasingly used across health research; however, it continues to be a complex area to navigate given existing institutional structures.

**Main body:**

We collaborated across three independent co-produced research studies to share insights, reflections, and knowledge of our work in the fields of HIV, mental health, and disability research. We co-designed and delivered a three-hour online workshop at a conference to share these reflections using the metaphor of ‘building bridges’ to describe our co-production journey. We generated key principles of co-production from our different experiences working in each individual research project as well as together across the three projects. Our principles are to: (1) be kind, have fun and learn from each other; (2) share power (as much as you can with people); (3) connect with people you know and don’t know; (4) remain connected; and (5) use clear and simple language.

**Conclusion:**

We recommend that co-produced research needs additional funding, resource, and flexibility to remain impactful and ethical. Co-produced research teams need to be mindful of traditional power structures and ensure that the process is transparent, fair, and ethical. Addressing equality, diversity, and inclusion of traditionally underrepresented groups in research is essential as are the skills, expertise, and experiences of all members of the co-production team.

## Abstract (plain language summary)

Co-produced research occurs when a range of people, including researchers and people with lived experience of a topic work together on all stages of a piece of research, from design to publication, to improve understanding of a topic. This methodology has become increasingly popular in the field of health research, however – it can be difficult to undertake because of how health institutions are structured. As a team, we examined three co-produced studies (focusing on HIV, mental health, and disability research) to share our insights and reflections. We then shared these reflections through a co-designed online workshop at a conference. We used the metaphor of ‘building bridges’ to describe our teamwork. From our experiences on these studies, we came up with five key suggestions for co-production in research: (1) be kind, have fun and learn from each other; (2) share power as much as possible with everyone; (3) connect with people you know and those you don’t; (4) stay connected; (5) use clear and simple language. To achieve ethical and impactful co-produced research, we suggest that it needs more funding, resources, and flexibility. Teams who are conducting co-produced research must be fair and clear about how they do so, and ensure that everyone, especially groups who are often unheard, get a chance to be part of research so that everyone’s skills and experiences are equally considered.

## Background

**Fig. 1:**
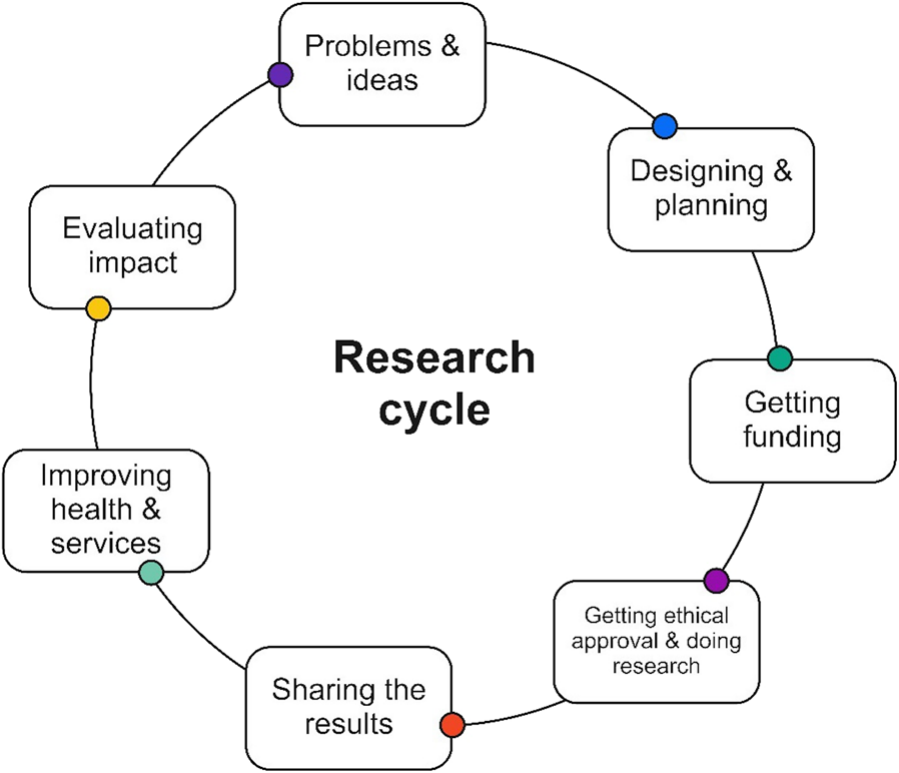
Co-production in the research cycle. Adapted from the NIHR [1]

Co-produced research is a partnership between academic researchers (academics), and people with lived experience (co-researchers) throughout the research cycle (Fig. 1). Specifically, this includes working together to identify research questions, collect and analyse information, write academic publications, and present findings to the public. Co-researchers may not have ‘formal’ research qualifications but rather insights and experiences deeply rooted in their everyday life and past histories.

In the United Kingdom (UK), the National Institute for Health and Care Research (NIHR) use a broad definition of public involvement of “research being carried out ‘with’ or ‘by’ members of the public rather than ‘to’, ‘about’ or ‘for’ them” [1]. Therefore, in the UK, co-produced research extends involvement beyond advisory and guidance-centred activities of patient and public involvement. As a result, co-produced research is a fair and ethical approach to conducting meaningful, empowering, and inclusive publicly funded research. This approach to research is sometimes categorised under the umbrella term of ‘participatory research’ [2]. These terms are often used interchangeably and with other types of approaches to involving people with lived experience in research; researchers have raised concerns about the misappropriated use of the term co-production [2, 3]. We use the term co-produced research as an approach to recognise and attempt to address and dismantle traditional power differentials in research to facilitate knowledge generation [4–8]. Power may not just be hierarchical but it can be intersectional too (or relate to the multiple identities people may hold e.g., relating to ableism, racism, class and education etc.) [9]. Co-produced research can be rewarding but also emotionally challenging for both academic researchers and people with lived experience particularly when involving populations who are already socially marginalised [7, 10, 11].

Here, we consolidate our lessons learned in this space using the ‘building bridges’ metaphor to share and reflect on our experiences at Imperial College London across three co-produced projects. Our projects, now complete, consisted of three teams of co-researchers: people living with different health conditions including HIV (in collaboration with peer-led HIV support charity Positively UK) [12–14], learning disabilities and autism (in collaboration with Heart n Soul) [5, 15–17] and young people with experience of mental health difficulties [18–20].

McMellon et al*.* [21] have reconceptualised co-production as “quiet activism”. This is pertinent in HIV, learning disabilities and mental health research whereby co-researchers have driven advances made in treatment, care and services by highlighting the stigmatisation and marginalisation experienced by people with lived experience.

## Methods

### Developing our collaborative work

**Fig. 2:**
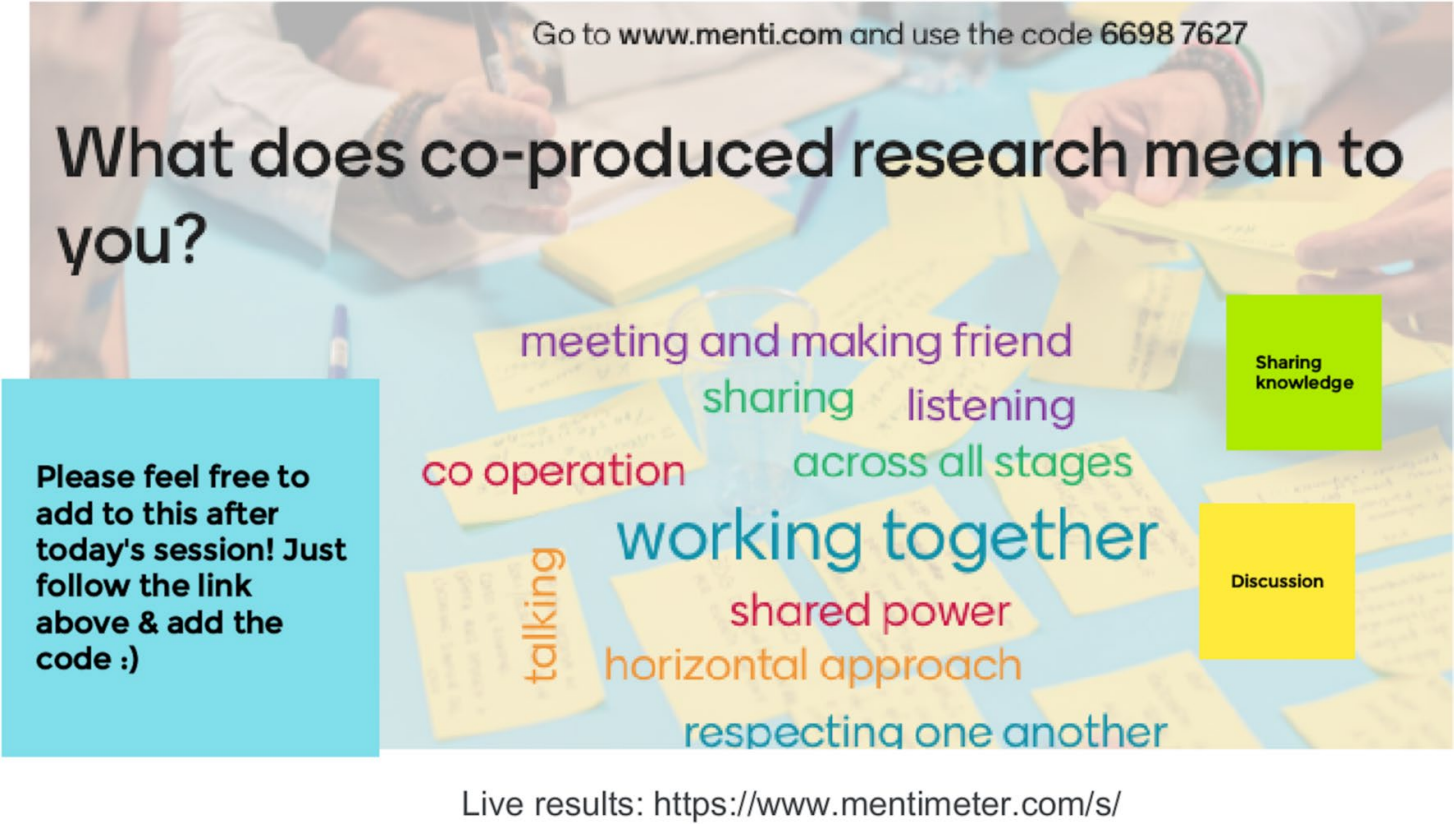
Mentimeter activity ran at first co-production planning meeting with co-researchers

VP identified DC and LD as two academic researchers with extensive co-production experience. We first met as a group in Autumn 2021 to develop a proposal submission to the National Centre for Research Methods (NCRM) e-Festival [22]. We were successful in securing a 3h online reflective workshop on co-production in research. We then brought a diverse group of six co-researchers together to design the workshop. We met twice online (Zoom) for 1.5h to get to know one another, design and develop the aims of the workshop, key roles, and responsibilities, and explore what co-produced research means to each person (Fig. 2). Co-researchers were renumerated for their preparation time and time co-presenting at the workshop in line with NIHR guidance [23].

**Fig. 3:**
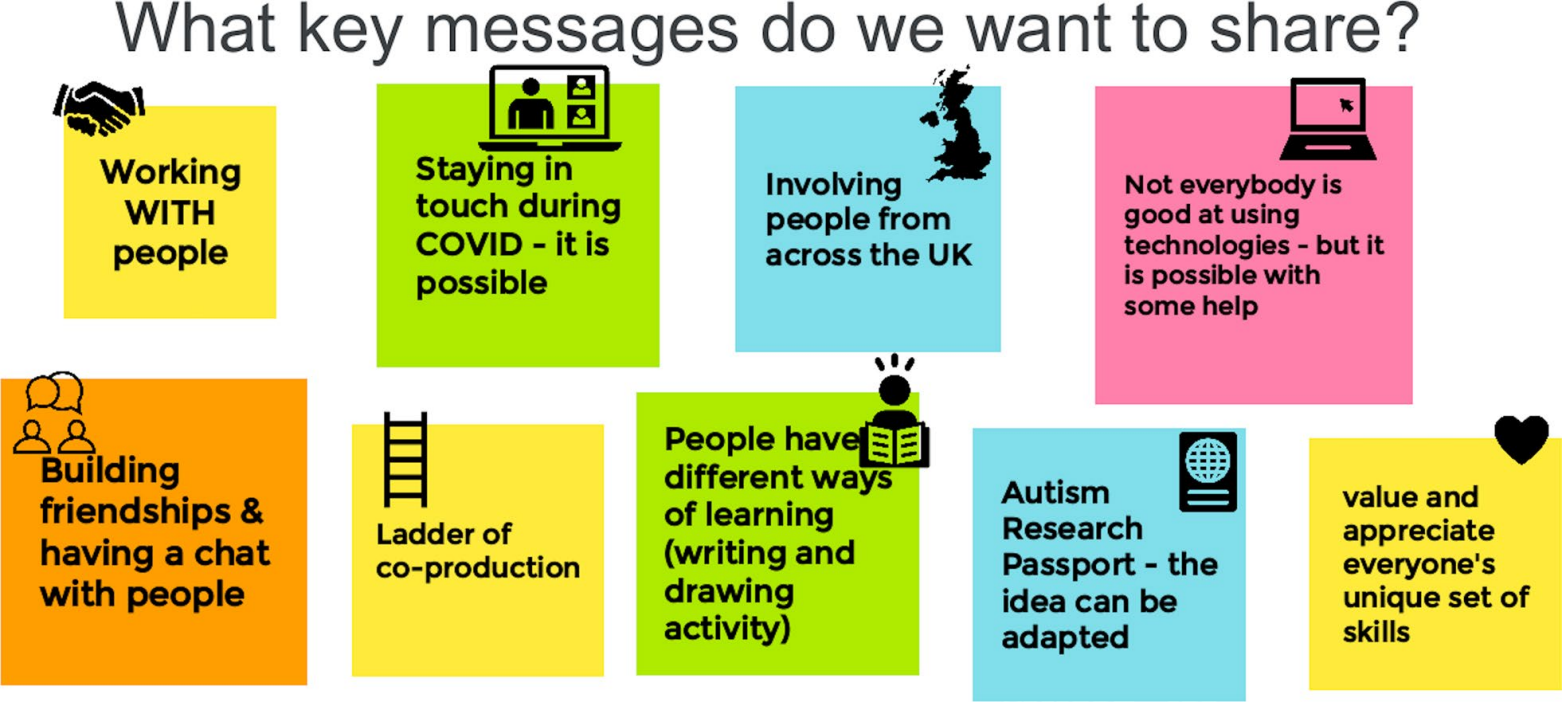
Our shared key messages for the NCRM e-Festival developed on Google Jamboard.

The planning meetings were facilitated by VP and we recorded discussions and ideas using Google Jamboard as all the research projects had successfully used this tool as a method for engagement [5, 12]. It was during these meetings that we also created shared principles for co-production designed by co-researchers from Heart n Soul and Positively UK. This included nine key messages to share which summarised into five key principles of co-production (Fig. 3) which were then used to guide facilitated discussions with co-researchers on their experiences of their involvement in each project. Following this initial collaborative work, VP, WT and JB also used these principles to guide the development of internal training for Imperial College researchers on participatory approaches:

1. Be kind, have fun and learn from each other
2. Share power (as much as you can) with people
3. Co-production can help you to connect with people you know (existing relationships) and don’t know yet (new relationships)
4. Remain connected
5. Use clear language/make things simple

By meeting teams representing different ‘lived experiences’, we discussed and challenged our own assumptions about current co-produced research practices; for example, the motivation behind co-producing research and how to make the process more accessible and equitable [24]. To develop this paper, we share our stories and reflections (as quotes) of conducting co-produced research using the metaphor of ‘building bridges’.

## Results

### Co-produced research is about ‘building bridges’

First, academics reach out and connect with underrepresented groups in research because of historical exploitative approaches when working with people with lived experience. In this process, academics try to *build a bridge* between universities and people who have been isolated from research practice. However, this step can also be vice-versa (communities or individuals reach out to academics) typically where already well-established and trusted relationships exist.

Once *the bridge is built*, academics and communities have access to each other and begin to develop relationships. Through an exchange of knowledge, experiences, practice and training, people with lived experience become co-researchers and the line between academics and co-researchers becomes blurred. However, this exchange can only happen if the bridging path is paved level for everyone. This means a path without unnecessary barriers, and with flexible time to cross, so everyone can access the bridge equally and walk at their own pace.

*“People have different kinds of ways to learn” (Pino, Co-researcher at Heart n Soul)*

*“If you are not learning, you are not engaging in the right way.” (Robyn, Co-researcher at Heart n Soul)*

Whether we are academics or people with lived experience (or both), we are all ‘new’ to some things and experts in others, and all have something unique to contribute to co-produced research. For our projects, we had expertise in the arts (Heart n Soul), activism and community mobilisation (Positively UK) and bringing fresh perspectives from lived experiences (young people with mental health difficulties).

### A learning experience: sharing space on ‘the decks’ and disrupting power dynamics

*“I think it’s so much down to building relationships and having the humility to come to know that we’re all experts, and we come together as experts, but we have different skills, and we learn from each other.” (Lindsay, Academic researcher)*

For co-produced research to be successful, we need to meet *halfway on the decks of the bridge*. If we work together in a safe and shared space, where everybody feels welcome and comfortable, we will all meaningfully contribute to the process. We need to be ready to ‘drop the baggage’ and move into a new space, that takes people out of their comfort zones. In this space, we must all be ready to negotiate power,

*“The bridge says something about power. We’re not in one place or the other and a shared sort of liminal spaces between the two.” (Jane, Academic researcher)*

Both co-researchers and academic researchers may feel uncomfortable with challenging traditional power structures. Tensions may arise due to discomfort which may initially be misinterpreted to be interpersonal difficulties [7]. Therefore, getting to know one another on a personal level can strengthen the partnership; this could be through sharing goals and aspirations of the project and research and understanding the motivations for getting involved. Meeting in informal spaces, encouraging engaging discussion and “having fun” all play an important role.

Academic researchers must challenge power structures that inhibit co-production and advocate for the principles of co-production in their research groups and institutions. For example, ensuring co-researchers are always paid for their time, have the equipment available to be involved and communicate in a way that works for co-researchers as individuals as well as a team (email, text, WhatsApp, telephone). Truly accessible co-production means shifting a ‘one-size-fits-all’ approach to a more bespoke model of working together.

### ‘Wobbly bridges’: navigating emotions and emotional labour

When co-produced research gains momentum, the aim is to “be kind, have fun and learn from each other.”^1^ However, there are hurdles to overcome while *crossing the bridge* that we have built. In fact, *the bridge can wash away* if there is no appropriate recognition that co-researchers are exposed to uncertainty. This is particularly true when the right tools are not available to embrace and manage that uncertainty as part of co-produced research.

*“Maybe it’s a series of connected bridges actually, that you’ve gone over one but actually your journey hasn’t finished, and you will encounter other bridges that have other challenges along the way. Like an archipelago?” (Jane, Academic researcher)*

While co-produced research mostly brings joy and fulfilment, sharing experiences with others may be emotional or ‘triggering’ at times and can impact all members of the team; the bridge may start to feel ‘wobbly’. Feelings of doubt and frustration mingle together with feelings of hope for making the world a better place for everyone [15]. Appropriate safeguarding, emotional support and well-being mechanisms need to be prepared for both the co-researcher and academics to ensure the research remains meaningful and ethical [5, 11]. This can also be supported through available supervision, mentoring, and coaching by peers and colleagues.

*“Of course, safeguarding the participants and co-researchers is crucial in mental health research but it’s also important to look after the academic. Bouncing things off with a colleague who is also a clinician, and sharing the concerns I have, has been really reassuring” (Lindsay, Academic researcher)*

If you intentionally *“give space for other people to speak” (Pino)* and *“listen to people how they feel” (Pino)* then the caring connection between people can act like *‘a glue’ or ‘cement’*. This can strengthen the bridge and make the teamwork stronger together during the inevitable ‘wobbles’; a safe space needs to be created to allow for feelings of doubt and frustration to be shared. Getting to know one another on an emotional level can help to strengthen team bonds. Having a trained professional (e.g., counsellor, peer support worker) as part of the team can help troubleshoot any serious concerns and ensure that somebody is available to support everyone involved.

The co-author academics (VP, LD, JB, DC) are all trained in qualitative research. This person-centred discipline has equipped us with the right tools to perform co-produced research; for example, active listening, reflexivity, flexibility, and acknowledging the complexities of knowledge creation. At our core, both academics and the co-researchers have similar values and working approaches (empathetic, caring and kind people) with co-researchers, which helps to *develop and sustain the bridge* [25].

### Building multiple bridges on the way

*“Once the bridge [the relationships] had been built, then it felt more like my (previous) experiences of doing this type of research (qualitative) and, looking ahead, it’s like the research I want to continue doing. So, it was going from past research experiences to future research aspirations.” (Vas, Academic researcher)*

With the right infrastructure and support [26, 27], co-researchers grow in confidence meaning *the landscape of the bridge changes*. There are multiple bridges and obstacles to overcome. The initial *co-produced research bridge* changes into a *bridge that connects* a co-produced research team with new opportunities, places, and communities.

The aim of co-produced research is for co-researchers to build capacity and support the co-researcher’s skill development. Co-researchers have a greater sense of ownership and they independently build further bridges. For example, several co-researchers across our teams are undertaking PhDs, working in the health and care sector, have presented their work at academic conferences [14] or have become involved in further community-based projects. Heart n Soul co-researchers are developing a radical approach to designing services with people with learning disabilities or autism [28].

*"The reason we are successful is because we know what the problem is" (Pino, Co-researcher at Heart n Soul)*

*“My experience of being involved in the project has been that the co-researchers bring an insider view/perspective of the research and that can only be a win for any research.” (Wezi, Co-researcher at Positively UK)*

## Conclusion

Throughout all our projects, we faced similar challenges relating to resources and time, navigating power structures and institutional processes which are often cited in the literature [29–31]. For example, although we used the speakers fee from the conference organisers to cover payment for our co-researchers, this was not enough. Therefore, we had to ‘top up’ funding through existing grants. An additional challenge is the funding of co-producing the dissemination of study results as this often occurs much later than research project funding allows.

Transparency in decision-making, inclusivity and fairness of opportunities is key to ensuring that co-produced research also upholds ethical standards and good practice [32, 33]. Therefore, to ensure co-produced research is conducted to a high standard and provides maximum opportunity and support to all involved, we recommend:

1. Increased funder commitment to resources and flexibility in reviewing funding applications (e.g., specific funding streams to spring-board participatory research led by early-career researchers and community groups as well as removing requirements for co-researchers to have institutional affiliations).
2. Flexible application and review processes by ethics committees for participatory projects (e.g., fast-tracked amendments following co-researcher involvement to prevent unnecessary delays in projects) [1].
3. A greater emphasis on co-authorship and perspectives of community members by publishers [5].
4. Increased capacity, training resources and support by professional bodies to conduct co-produced research.

Building on our growing experiences, we aim to champion co-produced research as a method within public health research and ensure that it is understood and valued within academia. Research communities globally are starting to take action to reignite co-produced research and subsequently ensuring equality, diversity, and inclusion are a central focus for all in health, social care and beyond. However, we still have a way to go. For a more creative and engaging explanation of ‘co-produced research’ and how it can be achieved, we invite you to watch two videos produced by our teams [34, 35].

## Data Availability

Not applicable.

## Abbreviations

NCRM: National Centre for Research Methods
NIHR: National Institute for Health and Care Research
UK: United Kingdom

## Footnotes

1 This was one of the co-production principles we developed as a group **(Fig. 3).**
